# Competition between Delta and the Abruptex domain of Notch

**DOI:** 10.1186/1471-213X-8-4

**Published:** 2008-01-21

**Authors:** Zifei Pei, Nicholas E Baker

**Affiliations:** 1 Department of Molecular Genetics, Albert Einstein College of Medicine, 1300 Morris Park Avenue, Bronx, NY 10461, USA

## Abstract

**Background:**

Extracellular domains of the Notch family of signalling receptors contain many EGF repeat domains, as do their major ligands. Some EGF repeats are modified by *O-*fucosylation, and most have no identified role in ligand binding.

**Results:**

Using a binding assay with purified proteins in vitro, it was determined that, in addition to binding to Delta, the ligand binding region of Notch bound to EGF repeats 22–27 of Notch, but not to other EGF repeat regions of Notch. EGF repeats 22–27 of *Drosophila *Notch overlap the genetically-defined 'Abruptex' region, and competed with Delta for binding to proteins containing the ligand-binding domain. Delta differed from the Abruptex domain in showing markedly enhanced binding at acid pH. Both Delta and the Abruptex region are heavily modified by protein *O*-fucosylation, but the *split *mutation of *Drosophila *Notch, which affects *O*-fucosylation of EGF repeat 14, did not affect binding of Notch to either Delta or the Abruptex region.

**Conclusion:**

The Abruptex region may serve as a barrier to Notch activation by competing for the ligand-binding domain of Notch.

## Background

The *Notch *mutation in the fruit fly *Drosophila *was the first mutation of embryonic development ever described. *Notch *mutant embryos die from neural hyperplasia because *Notch *is a negative regulator of neurogenesis[1,2]. In mammals, Notch signaling is involved in a wide array of other developmental processes including somitogenesis, angiogenesis, germ cell proliferation, immune development, axonal pathfinding, proliferation, and intestinal patterning [3-6]. Notch signaling probably contributes to the development, homeostasis and pathology of most organs [7].

**Figure 1:**
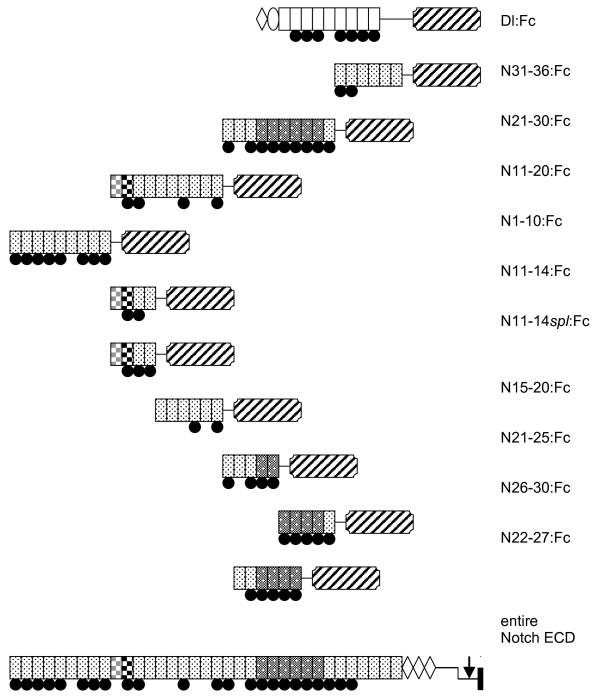
**Proteins expressed in this study**. The entire N ECD structure is shown at bottom, for comparison to the expressed proteins arranged above. Rectangular blocks represent EGF repeats and shaded circles, predicted *O*-fucosylation. Delta contains two domains, NT and DSL, conserved amino-terminal to its 9 EGF repeats. EGF repeats 11–12 from Notch, constituting the Ligand-Binding domain, are checquered. All Notch EGF repeats are shaded, and EGF repeats 24–29, affected by Abruptex mutations, are shaded darkly. The Fc domain (striped) is carboxy-terminal in all cases. Similar proteins lacking the Fc domain were also expressed (not shown). The LIN-12/N repeats are  represented by three rhomboids, disulfide bonds linking the two Notch chains by a vertical line, the ligand-sensitive S2 site by an arrow, and the trans-membrane domain by a solid bar.

Notch proteins are conserved cell surface receptors. Both *Drosophila *Notch and human Notch1 have extracellular domains containing 36 tandem EGF repeats, as well as other sequences[8]. A ligand binding domain comprising the two EGF repeats 11 and 12 has been defined using a cell adhesion assay. In this assay, *Drosophila *cells transfected to express Notch derivatives adhere to cells transfected to express either of two similar transmembrane ligands, Delta or Serrate, only if Notch EGF repeats 11–12 are present[9]. Genetic studies of Notch mutant flies confirm the importance of the EGF repeat 11–12 region in vivo[10]. EGF repeats 11–12 are far distant from the S2 proteolytic cleavage site that is thought to be the ligand-dependent step in Notch activation (Figure 1). The juxtamembrane S2 site is protected by a structure involving 3 Lin-12/N repeats that lie C-terminal to the EGF repeats [11]. How ligand binding at EGF repeats 11–12 is communicated to these distant regions of N is not known.

Little is known about the contribution of most of the EGF repeats to Notch function. The EGF repeat regions of Notch are predicted to form several rod-like arrays due to Ca-coordination by the inter-repeat linkers that precludes flexibility between repeats, with occasional linkers that do not bind Ca perhaps allowing localized flexibility[12]. The overall tertiary structure, and whether it involves interactions between inflexible EGF repeat regions, is not known.

Mutations affecting the Notch extracellular region point to functional importance for many EGF repeats in addition to the minimal ligand binding domain[13,14]. Similarities between Notch from *Drosophila*, humans, and other species further suggests functional conservation of EGF repeat regions. Some of these regions might interact with other ligands, or affect receptor clustering [15-20]. It has also been suggested that some EGF repeats might interfere with ligand binding[21].

Many EGF repeats are the substrate of an enzyme, OFUT-1, that that transfers fucose to Ser or Thr residues preceeding the third Cys of EGF repeats. The C^2^X_4–5_(S/T)C^3 ^consensus is found on 23 of the 36 EGF repeats of N, and 7 of the 9 EGF repeats of Dl[22]. In addition, the *O*-fucosyltransferase 1 enzyme acts independently of its catalytic activity, as a chaperone or trafficking factor [23,24]. A specific mutant allele of Notch, called *split*, is caused by an I578T substitution in EGF repeat 14 [25,13]. *split *results in increased Notch activity in some tissues, and introduces a site for *O*-fucosylation where one is normally lacking [26].

A specific allele of *Dl *that is a dominant suppressors of *spl*, called *Dl*^*sup*5^, corresponds to the G305R substitution[27]. It has been suggested that this mutation might prevent or reduce *O*-fucosylation of EGF repeat 3 on Thr306[27,28]. This suggested increased fucosylation of Notch on EGF repeat 14 might be suppressed by reduced fucosylation of repeat 3 of Delta. We noticed that *O*-fucosylation sites are predicted on many EGF repeats of Delta, but few on EGF repeats 11–20 of *Drosophila *Notch.

We wondered whether other EGF repeat protein regions might share properties with Delta. Like Delta, many of EGF repeats 22–30 from N bear *O*-fucosylation sites. This 'Abruptex' region of Notch is known for dominant mutations that affect EGF repeats 24–29 and which activate Notch signaling[13,25,29-32].

Here we used in vitro binding studies to detect specific binding between the ligand-binding region of *Drosophila *Notch and other EGF-repeat protein regions. Both Delta and the Abruptex region of Notch showed high affinity binding. We did not identify any effect of the *split *mutant, but the studies suggest that the Abruptex domain could oppose Notch activation by competing with Delta for the ligand binding site.

## Results

### In vitro interaction between extracellular domains of Notch and Delta proteins from *Drosophila*

An in vitro binding assay was explored as a means to detect interactions between EGF repeat regions of Notch and its ligand Delta. Hirai's group found that purified extracellular domains of mouse Notch2 and its ligand Jagged2 could interact independently of the cell surface when Notch2 extracellular-domains were adsorbed to an ELISA plate, and Jagged2 extracellular domains expressed as ligand-Fc fusion proteins[33]. The Fc domain from human IgG dimerizes the ligand, also serves as an affinity tag for purification, and is readily detected using secondary antibodies. The method is semi-quantitative and unlike cell surface expression experiments is less affected by variation in transgene expression level since the extracellular domains are partially purified before use[33,34].

We tested whether similar methods could detect interaction between *Drosophila *Notch and its ligand *Delta*. *Drosophila *Schneider cells were transfected to express secreted portions of the extracellular domains of Dl and Notch, tagged with a His_6 _tag and, where appropriate, the Fc domain from a human IgG (see Materials and Methods). All proteins expressed and purified in the course of our experiments are listed in Figure 1.

**Figure 2:**
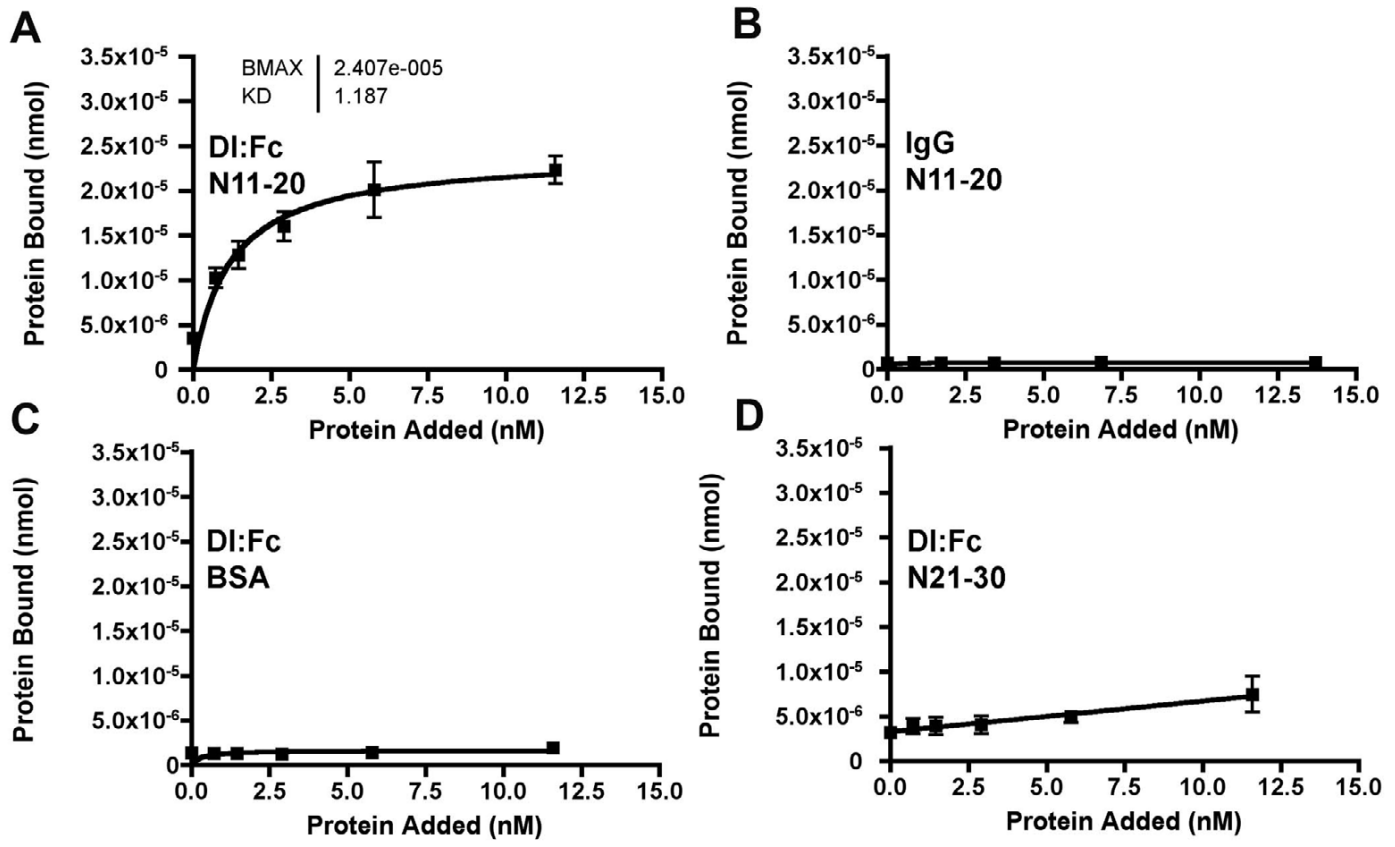
**Binding of Dl:Fc to a Notch protein including EGF repeats 11–12**. A. Dl:Fc binding to adsorbed N11-20, measured using HRP-conjugated anti-human IgG. In these and all subsequent experiments, error bars are standard deviations of parallel replicate experiments. Note that binding is saturable and therefore likely to be specific. Saturation binding and "Kd" (half-maximal binding) were estimated by the Prizm software package. B. Human IgG bound negligibly to N11-20, indicating that Dl sequences were responsible for most binding seen in panel A. C. Dl:Fc did not bind adsorbed BSA, so most of the binding in panel A was to adsorbed N11-20:V5:His. In subsequent figures, non-specific BSA binding by Fc fusion proteins has been measured and subtracted, so that binding specific to adsorbed N proteins is presented. D. Dl:Fc did not bind specifically to N21-30, another EGF repeat protein of equal length to N11-20, but lacking the known ligand binding site. Note that the small amount of signal detected is mostly present in the absence of added Dl:Fc protein, is not saturable, and is therefore likely to be non-specific.

To test whether purified extracellular domains of Dl and N from *Drosophila *could interact, we assessed the binding of purified Dl:Fc fusion proteins to V5-tagged, His-tagged EGF repeats 11–20 of the N extracellular domain. Henceforth we refer to this protein as N11-20. Figure 2 shows binding data for the interaction between Dl:Fc and N11-20 or N21-30. Fc-tagged proteins were detected using HRP-conjugated anti-Fc antibodies and a colorimetric assay.

Dl:Fc showed saturable binding to the N11-20 protein that contained the known Dl-binding site at EGF repeats 11–12 (Figure 2A). Human IgG alone bound negligibly to N11-20 (Figure 2B). Therefore, Dl sequences were responsible for the binding by Dl:Fc. Specific binding depended on the N11-20 protein, and was not evident when only BSA carrier was bound to the plate (Figure 2C). Dl:Fc also did not bind specifically to a N21-30 protein, which contained the same number of EGF repeats as N11-20 but lacked the known Dl-binding site (Figure 2D). Thus, in this assay Dl:Fc bound a portion of Notch that contain the Dl-binding site, and not to another EGF repeat portion of Notch that lacked the ligand binding domain.

In the experiment shown in Figure 2, Dl:Fc binding was half-maximal at 1.87 nM, compared to 0.7 nM measured for Jagged:Fc binding Notch2 in similar experiments[33]. It should be noted that half-maximal binding data do not correspond to true Kd values, because the stoichiometry of binding is unknown and because these may not be equilibrium binding measurements. In addition, whereas measurements were so reproducible in the short term that many error bars are invisibly small, they varied over weeks, perhaps depending on purity and storage of protein preparations. Therefore, quantitative comparisons have been made only between simultaneous, parallel experiments.

In the experiment shown in Figure 2A, Dl:Fc binding to 200 ng N11-20 protein saturated at 2.407 × 10E-5 nmol of Dl:Fc, 5.3% of the 4.56 pmoles of adsorbed N11-20. The stoichiometry of Dl/N binding is not known, but seems unlikely to be 1:20. More likely is that much of the adsorbed Notch is not available for binding, a typical finding when protein is adsorbed in non-oriented fashion.

In all remaining experiments, specific binding data are presented from which measured binding to BSA has been subtracted, although such background binding was always low.

### EGF repeat *O*-fucosylation and its contribution to binding specificities

**Figure 3:**
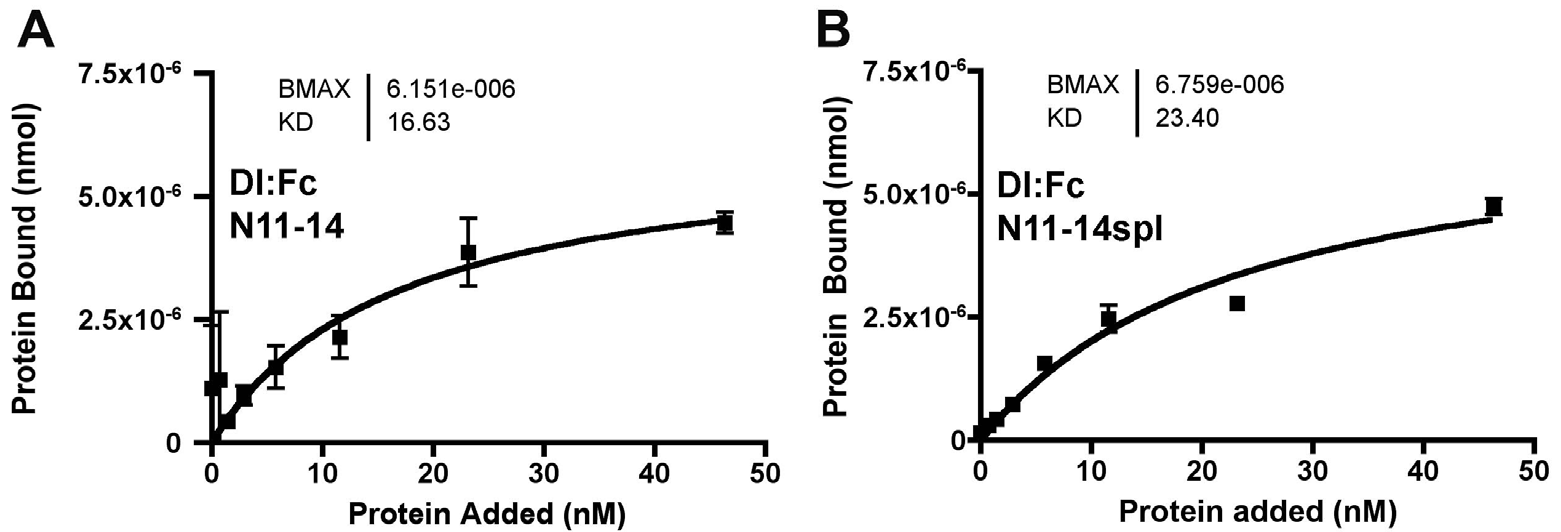
**Binding properties of the *split *mutant**. A. Dl:Fc showed saturable binding to adsorbed N11-14:V5:His protein, containing the ligand-binding site. B. Dl:Fc bound similarly to adsorbed N11-14*spl*:V5:His protein, containing an additonal site for *o*-fucosylation.

To assess the effect of the *split *mutation, N11-14 proteins were prepared with either wild type sequences or containing the I578T change from the *spl *mutation that inserts an *O*-fucosylation site on EGF repeat 14 (Figure 1). Previous studies show that the T578 mutant is *O*-fucosylated in *Drosophila *S2 cells [26]. Like N11-20, the N11-14 protein was bound by Dl:Fc. The I578T substitution had little effect on binding to Dl:Fc(Figure 3). Thus, the *spl *mutation appears not to affect N function through a direct effect on Dl binding, consistent with EGF repeat 14 not being part of the known ligand binding site[9].

### Direct interactions between the ligand-binding region and other regions of the N extracellular domain

EGF repeat 14 might be involved in binding to proteins other than Dl. Other regions of the Notch extracellular domain contains EGF repeats similar to Delta, and might be candidates.

**Figure 4:**
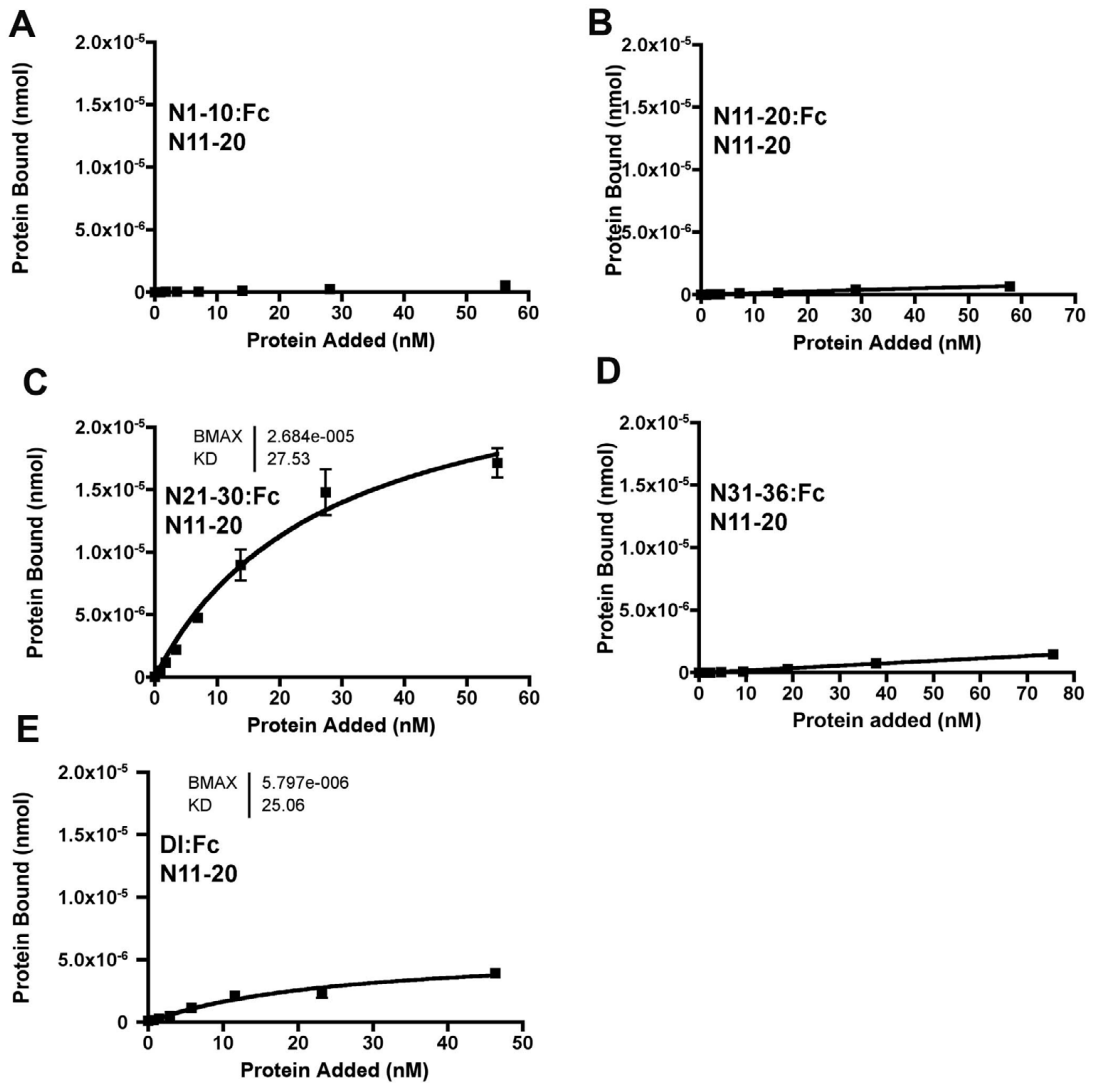
**Specific binding of N21-30:Fc to a Notch protein including EGF repeats 11–12**. A. N1-10:Fc did not bind to N11-20. B. N11-20:Fc did not bind to N11-20.C. N21-30:Fc showed saturable binding to N11-20. Although results vary from one protein batch to the next, in most cases more N21-30:Fc binds than does Dl:Fc (see panel E). D. N31-36:Fc did not show saturable binding to N11-20. E. Dl:Fc binding to N11-20.

To test whether other EGF repeat regions from N might interact with the ligand binding region, N1-10:Fc, N11-20:Fc, N21-30:Fc and N31-36:Fc proteins were assessed for interaction with N11-20 in vitro. N1-10:Fc, N11-20:Fc, and N31-36:Fc proteins showed negligible specific binding to N11-20 (Figure 4A–B,D). By contrast, N21-30:Fc showed saturable binding to N11-20 (Figure 4C). Although the absolute levels of binding varied between experiments, saturation binding to N11-20 was often higher for N21-30:Fc than for Dl:Fc (Figure 4E).

**Figure 5:**
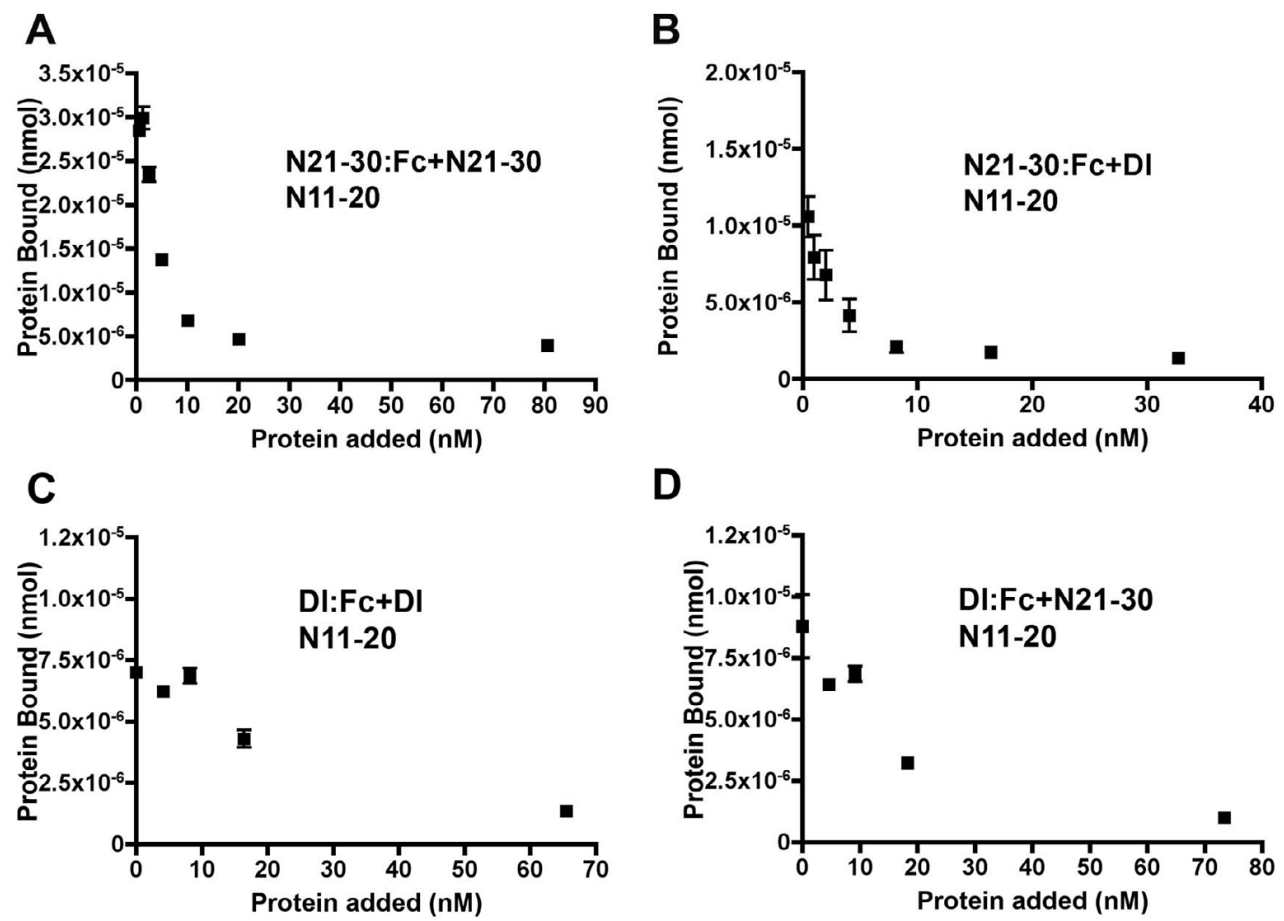
**Competition between Dl and the Abruptex region**. A. Binding of 10 ng (2.74 nM) N21-30:Fc to N11-20 is competed by increasing amounts of N21-30:V5:His. B. Binding of 10 ng (2.74 nM) N21-30Fc to N11-20 is competed by increasing amounts of Dl:V5:His. As Dl does not bind to N21-30 (see Figure 2D), Dl:V5:His binding to N11-20 is the likely mechanism. C. Binding of 100 ng (23.2 nM) Dl:Fc to N11-20 is competed by increasing amounts of Dl:V5:His. D. Binding of 100 ng (23.2 nM) Dl:Fc to N11-20 is competed by increasing amounts of N21-30:V5:His. As Dl does not bind to N21-30 (see Figure 2D), Dl:V5:His binding to N11-20 is the likely mechanism.

If N21-30:Fc and Dl:Fc both interact with N11-20 through the same binding site, then we would expect competition for this common site. To test this, soluble Dl:V5His and N21-30:V5His proteins were prepared and added as competitors. Despite the fact that N21-30 and Dl proteins were expected to be monomeric, but Fc-tagged proteins are dimers[33], N21-30 competed for N21-30:Fc binding, and Dl competed for Dl:Fc binding (Figure 5A,C). In addition, Dl competed for N21-30Fc binding (Figure 5B), and N21-30 competed for Dl:Fc binding (Figure 5D). Thus, purified Dl and N21-30 appeared to interact with N11-20 at the same or overlapping sites.

### EGF repeat *O*-fucosylation and its contribution to Abruptex binding

We hypothesized that although the I578T mutation on *split *did not affect interactions with Delta, it might affect interactions with N21-30. In the intact N molecule, such an effect could alter binding with Dl indirectly, if Dl competes with N21-30 for access to the ligand binding domain. Such a model could account for the suppression of the *spl *mutation by *Dl*^*sup*5^.

**Figure 6:**
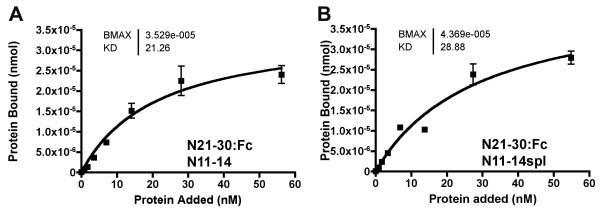
**Abruptex binding to the *split *mutant**. A. N21-30:Fc showed saturable binding to adsorbed N11-14:V5:His protein. B. N21-30:Fc bound similarly to adsorbed N11-14*spl*:V5:His protein.

The effect of the I578T mutation on interactions with N21-30 and other Notch regions was assessed. Like N11-20, the N11-14 and N11-14*spl *proteins were each bound by N21-30:Fc, but not by N1-10:Fc, N11-20:Fc, or N31-36:Fc (Figure 6A and data not shown). There was no discernible effect of the I578T substitution on the binding (Figure 6B). Thus, N11-14 and N11-14*spl *each behaved similarly to N11-20, without any discernible effect of the altered fucosylation on binding interactions. Additional experiments used artificial, four-EGF repeat proteins with complementary patterns of predicted *O*-fucosylation sites (see Materials and Methods). Such artificial proteins bound equuivalently to Dl:Fc and N21-30:Fc, and their complementary pattern of *O*-fucosylation sites did not confer specific affinity for one another (data not shown).

**Figure 7:**
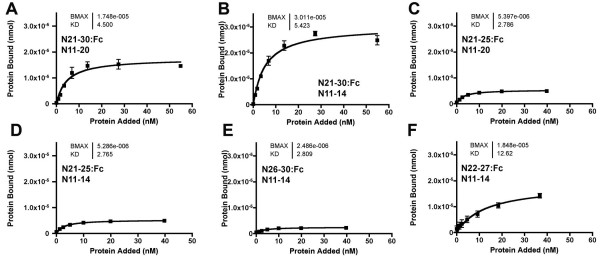
**Interactions between smaller proteins**. A. N21-30:Fc binding to adsorbed N11-20:V5:His protein. B. N21-30:Fc binding to adsorbed N11-4:V5:His protein. Saturation binding is increased compared to binding to adsorbed N11-20:V5:His protein (compare panel A), because 200 ng N11-14:V5:His protein is a greater molar amount than to 200 g N11-20:V5:His protein. C. N21-25:Fc shows reduced binding to N11-20:V5:His. D. N21-25:Fc shows reduced binding to N11-14:V5:His. E. N26-30:Fc shows little binding to N11-14:V5:His. F. N22-27:Fc shows saturable binding to N11-14:V5:His.

Smaller proteins have been examined to begin to define the specificity for Abruptex-region interactions. Figures 7A and 7B show that EGF repeats 11–14 contain the binding site for N21-30:Fc. EGF repeats 15–20 show little interaction (data not shown). EGF repeats 11–20 and EGF repeats 11–14 interacted quite poorly with both N21-25:Fc and N26-30:Fc (Figures 7C–E). The overlapping protein N22-27:Fc bound to EGF repeats 11–14 better, consistent with the major binding site(s) lying within the EGF repeats 22–27 region (Figure 7F).

### pH dependence of interactions

**Figure 8:**
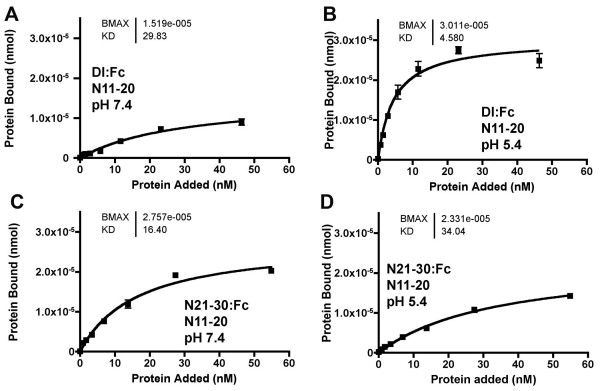
**pH effects**. A. Dl:Fc bound to adsorbed N11-20:V5:His protein at pH7.4. B. Dl:Fc binding to adsorbed N11-20:V5:His protein was significantly enhanced at pH5.4. Maximal binding was increased, 'Kd' was reduced. C. N21-30:Fc bound to adsorbed N11-20:V5:His protein at pH7.4. D. N21-30:Fc bound similarly to adsorbed N11-20:V5:His protein at pH5.4. In this experiment, maximal binding was slightly reduced, and 'Kd' was slightly increased.

**Figure 9:**
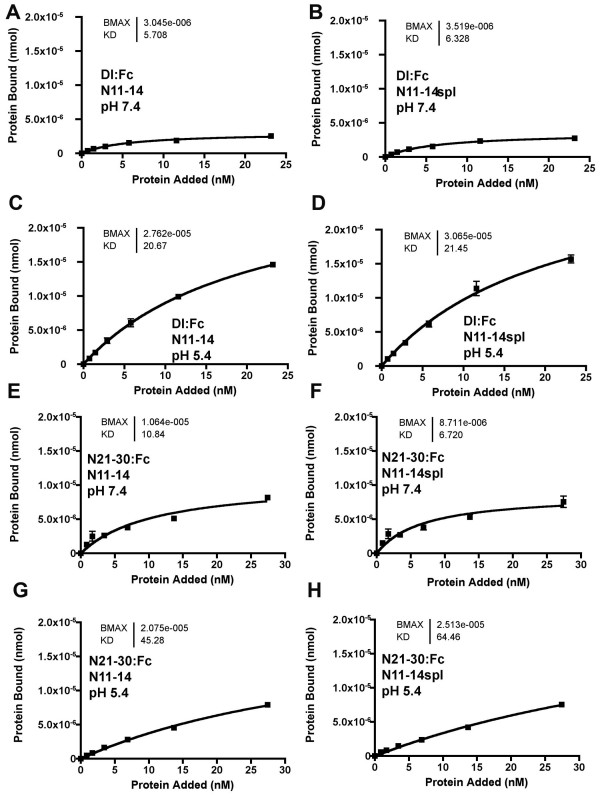
**pH does not affect binding to the *spl *mutant**. A. Dl:Fc bound to adsorbed N11-14:V5:His protein at pH7.4, B. Dl:Fc bound similarly to adsorbed N11-14*spl*:V5:His protein at pH7.4, C. Dl:Fc binding to adsorbed N11-14:V5:His protein was enhanced at pH5.4, D. Dl:Fc binding to adsorbed N11-14*spl*:V5:His protein was similarly enhanced at pH5.4, E. N21-30:Fc bound to adsorbed N11-14:V5:His protein at pH7.4, F. N21-30:Fc bound similarly to adsorbed N11-14*spl*:V5:His protein at pH7.4, G. N21-30:Fc binding to adsorbed N11-14:V5:His protein was not enhanced at pH5.4, H. N21-30:Fc bound similarly to adsorbed N11-14*spl*:V5:His protein at pH5.4,

In addition to the *Dl*^*sup*5 ^mutation, other second-site suppressors of *split *map in the *scabrous *and *gp150 *genes, which encode proteins found in late endosomes [35,36]. To better mimic the endosomal environment, the effect of low pH on interactions of the ligand binding domain was assessed. This required replacement of the Tris pH8.0 binding buffer. Either citric acid or Tris-maleate buffered binding solutions gave similar results at pH 7.4 to those described so far, and both these buffers were also usable at pH 5.4 (Figure 8A,C). Binding of Dl;Fc to N11-20 increased markedly at pH 5.4, both in terms of maximum binding, and apparent Kd (Figure 8B). No such increase was seen with N21-30:Fc binding to N11-20, which might be somewhat reduced (Figure 8D). To test whether *O*-fucosylation pattern affected interactions at low pH, Dl:Fc and N21-30:Fc binding was assessed with N11-14 and N11-14*spl *at different pH's. N11-14*spl *always bound to Dl:Fc or N21-30:Fc indistinguishably from N11-14, whether binding was performed at pH 7.4 or pH 5.4 (Figure 9).

**Figure 10:**
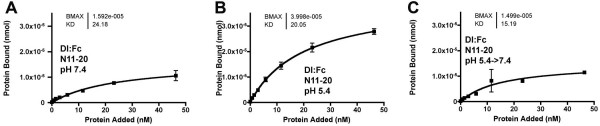
**Reversible pH effect**. A. Dl:Fc bound to adsorbed N11-20:V5:His protein at pH7.4, after 1 hour pre-incubation at pH7.4. B. Dl:Fc binding to adsorbed N11-20:V5:His protein was enhanced at pH5.4, after 1 hour preincubation at pH5.4. C. Dl:Fc binding to adsorbed N11-20:V5:His protein at pH7.4 was unaffected by 1 hour preincubation at pH5.4.

It has been suggested that ligands need recycling through an endocytic compartment for activation [37-39]. Our data raised the possibility that Dl might be activated by acidity during recycling. If this was the case, then we would expect that the 'activation' of DlFc observed at pH5.4 would be irreversible, and maintained after return to neutral pH. By contrast, if Dl:Fc interacts with N better at low pH, this will be reversed at neutral pH. To distinguish these possibilities, Dl:Fc was pre-incubated at pH 5.4 for 1 h, then half the sample was assessed for binding to N11-20 at pH 5.4, the other half neutralized and assessed for binding to N11-20 at pH 7.4 (Figure 10). Neutralized Dl:Fc bound to N11-20 less well than Dl:Fc at pH5.4, and not distinguishable from Dl:Fc protein that was never pre-incubated at pH 5.4 (Figure 10). The results indicate that low pH did not activate Dl:Fc irreversibly, but that pH made a direct, reversible contribution to the binding interaction.

## Discussion

In vitro studies were performed to explore how proteins interact with the ligand binding region of Notch, with three main findings: 1) the EGF repeat 21–30 region of Notch can compete with Dl for binding to the ligand binding region of Notch; 2) Such interactions between EGF repeat regions are not determined by *O*-fucosylation pattern; 3) The interaction of Dl with N is significantly enhanced at pH's typical of endosomes, more acidic than has previously been used to assess Notch function.

### The Abruptex domain competes with Delta binding

The EGF repeat 21–30 region of Notch bound to the Notch ligand binding region in vitro, and competed with the Dl extracellular domain for binding(Figures 5, 6). The binding regions were mapped approximately to EGF repeats 11–14 and EGF repeats 22–27, respectively (Figure 7). Other EGF repeat regions of Notch did not bind to the ligand binding region in this assay (Figure 4).

Another recent study has suggested that the Abruptex domain participates in Notch-Notch interactions, based on indirect evidence[21]. The specific interactions predicted are not the same as that which we directly demonstrate here. Our conclusions are conceptually similar, however. It should be noted that our experiments might not detect Notch-Notch interactions that required other stabilizing interactions in the context of the entire Notch molecule, or ternary complexes also involving Delta, should such interactions exist.

### *O*-fucosylation of the *split *mutant protein does not affect direct Notch-Delta binding

Historically, we sought to evaluate Notch-Notch interactions because of the *spl *mutation of Notch, and its suppression by the *Dl*^*sup*5 ^mutation[26]. If the *spl *mutation interfered with binding of Notch to the Abruptex domain more than to Delta, this could account for increased Notch activity, which could be suppressed by a further mutation of *Dl*. We did not detect any effect of the *spl *mutation on binding to Dl or to the EGF repeats 21–30 domain, however. Protein *O*-fucose is itself the substrate of Fringe glycosyltransferases, so that EGF repeats can be the carriers of a short oligosaccharide at this site[22]. Fringe modifies binding interactions and functions of Notch and its ligands, although not all Notch functions depend on Fringe [40,20-45]. It would be interesting to investigate whether modification of *O*-fucose groups by Fringe enzymes affects interactions between Notch domains, but we did not investigate this because in vivo the *spl *mutation affects Notch signaling independently of Fringe [26]. Although it is possible that *spl *does not affect protein-protein interactions of Notch, we cannot exclude an effect on an interaction not studied here, perhaps involving other proteins, should such interactions exist.

### pH effects on Notch-Delta interactions

Low pH favored interaction of Dl with N(Figure 8). The findings raise the possibility that Dl might displace Abruptex-domain more effectively after endocytosis. A role for endocytosis in Notch activation would be distinct from the now well-established requirements for Epsin, Neuralized, and Mind-bomb to endocytose Notch ligands, both because these latter molecules act in the ligand-producing cell, and because they are hypothesized to generate an active ligand recycled to the cell surface[46]. By contrast, we found the effect of pH on Dl to be reversible, and therefore not likely to affect ligands after recycling to the cell surface (Figure 10).

Although genetic evidence supports the idea that endocytosis is required in the receptor-activated cell in addition to the ligand-presenting cell[47], its specific role in Notch signalling is uncertain. It is not certain whether ligand-dependent Notch cleavage occurs inside the cell[8,48,49].

Dl is also believed to "cis-inactivate" Notch function when co-expressed in the same cells [50-53]. Recent studies indicate that cis-inactivation occurs at the cell surface, however, and that endocytosis is not required[54].

### Possible roles of the Abruptex domain in vivo

Could the Abruptex domain interact with the ligand binding domain in intact Notch molecules in vivo, as in in vitro experiments with protein fragments? Because mutations within the Abruptex domain enhance Notch signaling[13,25,29-32], it is reasonable to propose that the Abruptex domain normally interferes with Notch activation. Competition between the Abruptex domain and Delta for the ligand binding site of Notch provides a plausible mechanism by which this could occur. It has also been suggested that the Abruptex domain may be involved in cis-inactivation [31,32]. The structural basis for cis-inactivation is not known.

Notch activation is thought to require 'opening' of a receptor structure that protects the juxtamembrane S2 cleavage site [11]. It is not known how ligand binding to the EGFR11-12 region achieves this, because the S2 cleavage site is in the distant juxta-membrane region of the Notch extracellular domain, separated by the Abruptex domain and by other sequences (Figure 1). Ligand binding to the ligand-binding region could initiate a conformational change by displacing the Abruptex domain[21]. It is not known how this would deprotect the S2 cleavage site, however, especially as Notch molecules lacking EGF repeats 1–18 are not active[55]. One alternative model is that mechanical force unfolds the S2 region in response to ligand binding [11,56]

EGF repeats 11–14 and 22–27 from the same molecule seem unlikely to be in proximity if EGF repeats 10–21 form a stiff linear array, as is now thought[12]. Inter-molecular Notch-Notch interactions might be possible, however. Cell adhesion assays have not detected homophilic interactions between Notch proteins [57], but it is possible that Notch molecules on different cells cannot interact, for example if inter-molecular interactions have saturated before cells are mixed. In addition, Dl binding to N in such assays requires sequences amino-terminal to EGF repeats[58]. No such sequences are present in the EGFR21-30 segment of Notch, which was not able to replace the extracellular domain of Dl in cell adhesion and Notch signalling assays (our unpublished results). Should they occur, inter-molecular Notch-Notch interactions could cluster Notch molecules into dimers or into chains. It will be interesting to determine whether such structures occur and contribute to Notch regulation.

## Conclusion

We demonstrate binding in vitro between the Abruptex domain of the Notch extracellular domain and its ligand-binding domain, and propose that a similar interaction in vivo creates a barrier to Notch activation that is overcome by ligand binding,

## Methods

### Cloning

Plasmids expressing regions of the Notch or Delta extracellular domains were fused to a human IgG Fc fragment using a PCR strategy to clone open reading frames into an expression vector, pMT-Fc, that incorporates the amino-terminal signal sequence from the BiP protein. First, intronic sequences were removed from the Fc coding region. Two Fc exons were amplified from the pSecTag plasmid (Invitrogen) using two primer pairs: ccgtctgagacatgcccaccgtgccca/gaaggcctttggctttggagatggttttc and gctgcgcagccccgcgcaccaca/gctctagatttacccggagacagggagag. PCR products were digested by XhoI/StuI and FspI/XbaI respectively, then inserted into XhoI XbaI digested pMT/BiP/V5-His (Invitrogen) in a three-way ligation. Extracellular portions of Notch or Delta were expressed by amplification from the pMTN or pMTDL plasmid templates. PCR products were digested with EcoRI and XhoI and ligated with EcoRI and XhoI digested pMT-Fc. The primers for Notch were:

ggaattcgttggtggccgcttcctgcacaag – forward primer from Leu57 N-terminal to EGF repeat 1.

ggaattcgtcagaggacatagatgaatgcgat – forward primer from Ser447 N-terminal to EGF repeat 11.

ggaattcggagatcaatatcaacgattgc – forward primer from Glu600 N-terminal to EGF repeat 15.

ggaattcggaaacgaatattgacgactgt – forward primer from Glu827 N-terminal to EGF repeat 21.

ggaattcgcagacaaacgatgaggattg – forward primer from Gln1000 N-terminal to EGF repeat 26.

ggaattcggaactgaacatcgatgactgtg – forward primer from Glu1219 N-terminal to EGF repeat 31.

ccgctcgagttcatctatgtcctcgaacaatcc – reverse primer to Glu452 C-terminal to EGF repeat 10.

ccgctcgagatcgttgatattgatctcgcaac – reverse primer to Asp605 C-terminal to EGF repeat 14.

ccgctcgaggtcgtcaatattcgtttcgcac – reverse primer to Asp832 C-terminal to EGF repeat 20.

ccgctcgagatcctcatcgtttgtctgacaattg – reverse primer to Asp1025 C-terminal to EGF repeat 25.

ccgctcgaggtcatcgatgttcagttcgcaattg – reverse primer to Asp1224 C-terminal to EGF repeat 30.

ccgctcgaggttggcatcatagatatcgcag – reverse primer to Asp1456 C-terminal to EGF repeat 36.

The primers for Delta were:

ggaattcgcaggttcacagttcacagttccggcagc – forward primer from Gln19.

ccgctcgagctcatcgcactgcttgcccc – reverse primer to Glu560.

Plasmids expressing regions of the Notch or Delta extracellular domains tagged with V5-6His were constructed by ligating pMT/BiP/V5-His instead of pMT-Fc.

To replace Ile464 with Thr in EGF repeat 11 from Notch, the oligonucleotide ggcctACCtgcgtgaacacaccgggcag was used for oligonucleotide-mediatedmutagenesis. To replace Ser502 with Ile in EGF repeat 12 from Notch, the oligonucleotide cgggATCtgcctggatgatccggggaacg was used for oligonucleotide-mediatedmutagenesis. To generate fucosylated or un-fucosylated 4-EGF repeat proteins, EGF repeats 11–12 containing either a Thr464 mutations to introduce a fucosylation site to EGF repeat 11, or a Ile502 mutation of the fucosylation site on EGF repeat 12, were amplified and each cloned as tandem repeats. In the first reaction the forward primer (from Ser447) was gaagatcttcagaggacatagatgaatg, the reverse primer (to Asp526) was ccatcgatttcgcactgtgtgcccgtg. In the second reaction the forward primer (from Asp449) was ccatcgatatagatgaatgcgatcaggagtc, the reverse primer (to Glu529) wasccgctcgagttcgtccaatgtcgatttcgcac. The two PCR products were digested with BglII/ClaI and ClaI/XhoI respectively and subcloned into BglII/XhoI disgested pMT/BiP/V5 using a three-way ligation.

### Cell culture

Schneider cells were kept at 25°C in Shields and Sang M3 medium (Sigma) supplemented with 10% heat-inactivated fetal bovine serum (Sigma) and 50 U/ml penicillin, 50 U/ml streptomycin (Invitrogen). Cells were transfected using lipofectin as described [59]. Proteins were harvested from serum-free M3 medium 48 h after induction with 0.5 mM CuSO_4_. Secreted His-tagged proteins were purified from conditioned media using ProbondTM resin (Invitrogen), eluted in 50 mM NaH_2_PO_4_, 500 mM NaCl, 300 mM imidazole, pH7.5 and stored at -80°C prior to serial dilution in TBS or other buffers as described for use(TBS: 10 mM Tris-Cl pH 8.0 150 mM NaCl, 1 mM CaCl). Fc-Tagged proteins were purified using Protein A beads (Amersham), eluted with 0.1 M Glycine HCl pH 2.6 into 10 mM Tris pH 7.4 and stored at -80°C prior to serial dilution in TBS or other buffers as described for use. Purity and yield were assessed using a Protein-Assay Kit from Pierce and Coomassie Blue staining of SDS-PA gels. Fc-tagged proteins were usually recovered ~95% pure, His-tagged proteins ~85–90% pure. Storage for more than a few weeks reduced activity in binding assays, although no degradation was apparent.

### Binding Assay

200 ng of His-tagged protein eg N11-20V5H was added to each well of a 96-well plate (Apogent) in 50 μL TBS at 4°C overnight. After 3 TBS washes, the plate was blocked with 10 mM Tris-Cl (pH 8.0), 150 mM NaCl, 1 mM CaCl_2_, 3% BSA at 4°C overnight. After 3 washes with TBS containing 0.05% Tween20, known amounts of purified Fc fusion protein (eg Dl:Fc) were added in 50 microlitres of TBS, and after 2 h incubation the plate was washed 3x with TBS + 0.05% Tween20. After incubation with HRP-conjugated anti-human IgG antibody (1:5000 in TBS), the plate was washed three more times in TBS + 0.05% Tween20, and bound antibody detected with the HRP development reagent and quantified with a microplate reader (Perkin Elmer Wallac Victor2). Other pH values were achieved using citric acid/Na2HPO4 or Tris maleate/NaOH buffers in place of Tris pH8.0[60]. Tris maleate/NaOH appeared to show better Ca solubility.

### Binding curves

Data were compiled using Prizm 4 software (GraphPad). Errors (standard deviations from replicate experiments) are shown for *all *datapoints (but are too small to see in some figures). Molarities bound are expressed on a monomer basis.

## Authors' contributions

ZP carried out the experimental work, contributed to the data analysis and helped to draft the manuscript. NEB conceived of the study, participated in its design and coordination and drafted the manuscript. Both authors read and approved the final manuscript.
